# Fracturing in coals with different fluids: an experimental comparison between water, liquid CO_2_, and supercritical CO_2_

**DOI:** 10.1038/s41598-020-75787-y

**Published:** 2020-10-29

**Authors:** Jianfeng Yang, Haojie Lian, Li Li

**Affiliations:** 1 School of Energy Engineering, Xi’an University of Science and Technology, Xi’an, 710054 Shaanxi China; 2 Key Laboratory of In-situ Property-Improving Mining of Ministry of Education, Taiyuan University of Technology, Taiyuan, 030024 Shanxi China; 3 Civil and Environmental Engineering, University of Waterloo, Ontario, Waterloo N2L3G1 Canada

**Keywords:** Civil engineering, Coal, Natural gas, Energy science and technology

## Abstract

The present work conducted laboratory experiments of fracturing in fat coals, anthracites, and mudstones. Three different fluids were selected as the fracturing fluids, including water, liquid CO_2_ (L-CO_2_), and supercritical CO_2_ (Sc-CO_2_). The resulting fracture morphologies and fracture apertures of the coal specimens were investigated using 3D morphological scanning, and the permeabilities of the samples were measured before and after fracturing. The experimental results showed that the breakdown pressures of Sc-CO_2_ fracturing were the lowest among the three fracturing fluids, and the average single fracture apertures of the ScCO_2_-induced cracks were the smallest amongst the three fracturing fluids. In addition, the number of cracks and the roughness coefficients induced by Sc-CO_2_ fracturing were larger than those caused by water and liquid CO_2_. The viscosity of the fracturing fluid and the capillary effect are key factors that affect the crack propagation path and fracture surface topography. The results suggest that Sc-CO_2_ has the largest diffusion length, and thus is capable of permeating the coal matrix through small pores and causing more extensive fractures. Additionally, the effective hydraulic apertures of coal specimens produced by Sc-CO_2_ fracturing were wider than those induced by water and liquid CO_2_. The experimental results indicate that Sc-CO_2_ fracturing has huge potential to enhance coalbed methane recovery.

## Introduction

Coalbed methane (CBM) refers to hydrocarbon gases existing in coal seams, whose main component is CH_4_. The exploitation and utilization of CBM is of crucial importance to improving the supply of clean energy^1^, ensuring safe production in coal mines and reducing greenhouse gas emissions^2^. According to the national coal bed methane resources evaluation report of Ministry of Land and Resources, The amount of prospective resources within 3000 m is 55.20 trillion cubic meters in China, which is the third largest reserves in the world (less than Russia and Canada)^3^. Nevertheless, the efficiency of CBM exploitation in China has not yet reached to the expected level^4^. The cumulative CBM production was 17.1 × 10 m^3^ by the end of 2015, which is the only accounting for about 60% of the total target volume in the plan of the government^5^. One of the primary reasons restricting the efficient exploitation of CBM in China is the low permeability of coal seams (< 1 mD)^6^. Hydraulic fracturing is a commonly used approach for enhancing CBM production in low permeable coals, whose key idea is to inject a large amount of water into ground to create interconnected dense fracture networks^7^. Nevertheless, this technology is not without shortcomings. For example, it results in groundwater contamination by additives that cannot be degraded naturally in the aqueous fracturing liquid^8^. Another main obstacles with hydraulic fracturing is excessive water consumption^9^, which is even more serious considering the situation that the most CBM reservoirs in China are situated in water-deficient areas. In addition, some coal seams in China consist of mudstones interbedded with high clay content, and hydraulic fracturing causes the clays to swell and obstructs gas transport channels, incurring the so-called water block effect.

**Figure 1:**
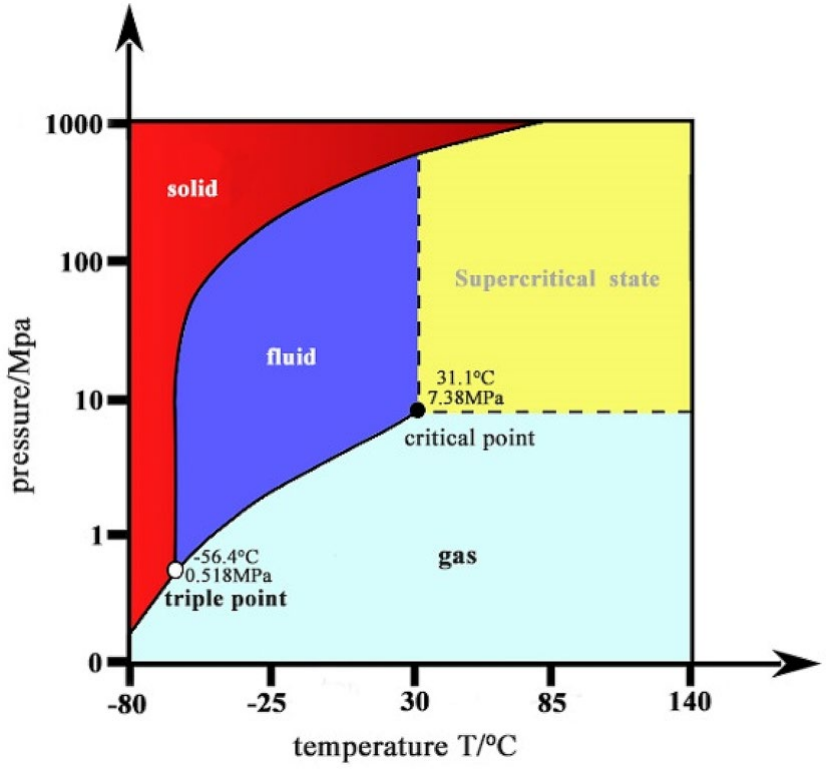
Phase transition diagram of carbon dioxide.

To surmount the above-mentioned drawbacks, the non-aqueous fracturing technologies are presented by some researchers^10–12^, in which carbon dioxide (CO_2_) is considered to be a competitive alternative to water. It is noted that although liquid CO_2_ (L-CO_2_) is normally injected as fracturing medium in engineering practice, it is highly possible to transit to supercritical state in deep coal seams, where the pressure and temperature exceed the critical point of CO_2_ (7.38 MPa and 31.1 °C) (Fig. 1)^13^. Supercritical CO_2_ (Sc-CO_2_) holds promise as an ideal fracturing medium for its low viscosity (similar to CO_2_ gas) and high density (similar to liquid CO_2_)^14^. Another benefit of Sc-CO_2_ fracturing is that it is able to displace the CBM existing as adsorbed form due to the higher adsorption properties of Sc-CO_2_ in coals^15,16^ and meanwhile reduces greenhouse gas emissions^17^.

Since its inception, the non-aqueous fracturing with L-CO_2_ and Sc-CO_2_ has drawn considerable attention^18,19^. Verdon et al.^20^ monitored the seismic wave variations which occurred when aqueous fluid and CO_2_ were injected separately into the reservoir. Their results showed that the distinguishable fracturing behaviors of water and Sc-CO_2_ arise from their entirely different physical properties. Ishida et al*.*^21^ compared waterless (including liquid CO_2_ (L-CO_2_) and Sc-CO_2_) and water fracturing in granite. Their experimental results indicated that, compared to water injection, the crack propagation ranges induced by L-CO_2_ and Sc-CO_2_ were greater and the breakdown pressures were considerably lower. Zhou et al*.*^22^ conducted Sc-CO_2_ fracturing experiments with cylindrical shale samples and used computerized tomography (CT) scanning to characterize the induced fractures. Their experimental results revealed that Sc-CO_2_ produced extensive and complex multi-branch fractures in the shale samples. Zhang et al*.*^23^ performed experiments on Sc-CO_2_ fracture initiation and the crack propagation behavior driven by Sc-CO_2_ in large shale specimens using acoustic emission (AE) and CT scanning systems, and found that the fracture initiation pressure caused by Sc-CO_2_ fracturing was about 50% less than that for water fracturing and that complex fracture networks were more likely to occur in the Sc-CO_2_ fractured samples. According to Jia et al*.*^24^, Sc-CO_2_ fracturing induced fractures with rougher and more complex surfaces and higher tortuosity, and increased the permeability of samples to a greater extent compared to water fracturing. Similar conclusions were drawn by Zhou et al*.*^25^ who carried out Sc-CO_2_ fracturing experiments on artificial rock specimens and Cai et al*.*^26^ who conducted Sc-CO_2_ jet fracturing tests on solvent glass specimens. Experimental investigation^27–30^ further revealed that the fracture toughness of coals tends to decrease with the injection of Sc-CO_2_, causing cracks in the coal to expand more easily. Parallel to the development in laboratory experiments, some advances are also made in mathematical modelling. Wang et al*.*^31^ adopted a coupled model of rock fracture mechanics and gas flow to simulate the water, oil, and Sc-CO_2_ fracturing processes, finding that Sc-CO_2_ fracturing exhibited the lowest breakdown pressure, which is consistent with the experimental results from other studies. Song et al*.*^32^ presented a multi-field coupling mathematical model to investigate the pressure transmission in the tubing used for Sc-CO_2_ fracturing, and Yang et al*.*^33^ developed a new numerical model to predict the evolution of temperature and pressure during Sc-CO_2_ fracturing. These mathematical models offer a theoretical basis for Sc-CO_2_ fracturing engineering.

The novelty of the present work lies in that we provide a systematic framework for assessing the performance of different fracturing fluids, in the perspectives of breakdown pressure, number of cracks, and particularly, fracture roughness coefficients and fracture apertures. We performed laboratory fracturing experiments using water, L-CO_2_, and Sc-CO_2_ as the fracturing fluids on coal core specimens obtained from the main CBM production areas in the Qinshui Basin, China. A triaxial fracturing apparatus designed in-house was employed in the experiments. The permeabilities of the specimens were measured before and after fracturing. Then, the fracture surfaces induced by the different injected fluids were scanned by the 3D morphological scanner and following this, the fracture roughness and apertures are quantified.

## Materials and methods

The laboratory experiments have undergone the following stages: first, fracturing experiments with the coal specimens were carried out using the different fluids (water, L-CO_2_, and Sc-CO_2_) under the same operation conditions. Second, after the fracturing the permeabilities of the coal core specimens were measured. Third, the surface topographies of the induced fractures in the coal specimens were studied to compare the fracturing performance of the different fluids. Finally, the fracturing effects were further compared between the three fracturing fluids and analyzed in detail to explain the underlying reasons.

### Materials used and sample preparation

**Figure 2:**
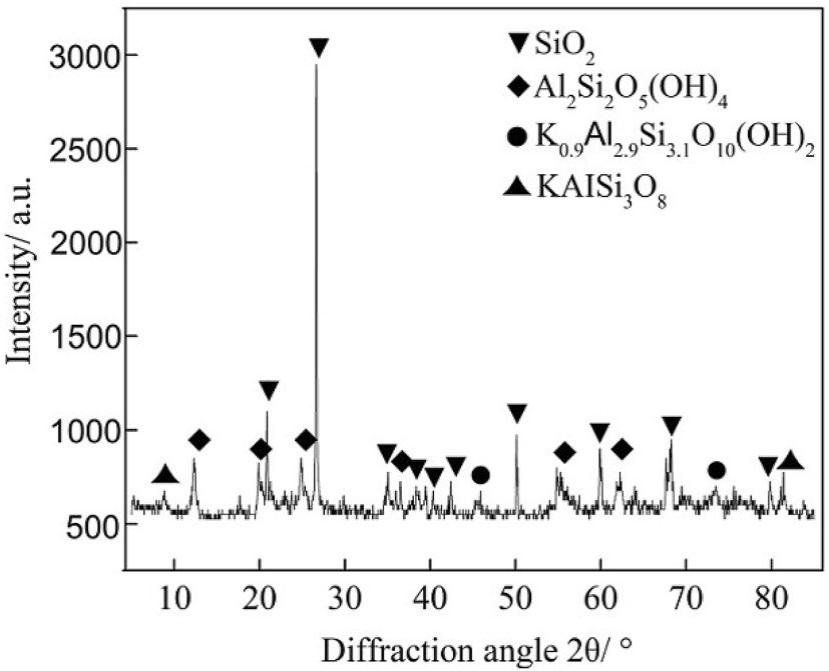
XRD spectrum of the mudstone used in the experiments.

Coal rank is a quantity used to describe the degree of coal mineralization in the coal formation process. Generally, coal is divided into anthracite (high-rank coal), bituminous coal (mid-rank coal) and lignite (low-rank coal) according to fixed carbon content, volatile matter, vitrinite reflection and other parameters, which bituminous coal includes long-flame coal, gas coal, fat coal, coking coal and lean coal^34^. In this study, two different rank coals were tested: a mid-rank fat coal and a high-rank anthracite, which were collected from the Changping and Hesi collieries located in the Qinshui Basin. This area is the most important CBM production base in China, accounting for over 66% of China’s total CBM production^35^. Proximate analysis results and mechanical parameters of the coal samples are listed in Table 1. Additionally, mudstone specimens taken from the Changping colliery were used as a control group. The mineral content of the mudstone samples was determined semi-quantitatively using X-ray diffraction (XRD), with a typical XRD pattern as shown in Fig. 2. The results show that the clay mineral content in the mudstone specimens was 34%, including kaolinite and illite, and the rest was composed predominantly of silicon dioxide and potash feldspar. The samples taken from the mine shaft were immediately sealed with paraffin wax to avoid weathering.

**Table 1:** Proximate analysis results and mechanical parameters of the coal and mudstone samples.

Type	Fat coal	Anthracite	Mudstone
Moisture content (%)	0.63	2.54	–
Ash yield (%)	7.90	19.37	–
Volatile matter (%)	30.35	8.02	–
Fixed carbon (%)	61.43	71.33	–
Vitrinite reflection (%)	1.12	2.53	–
Elasticity modulus *E* (MPa)	2750	3120	36,500
Poisson's ratio *ν*	0.28	0.29	0.32
Tensile strength *f*_t_ (MPa)	0.57	1.12	4.52

**Figure 3:**
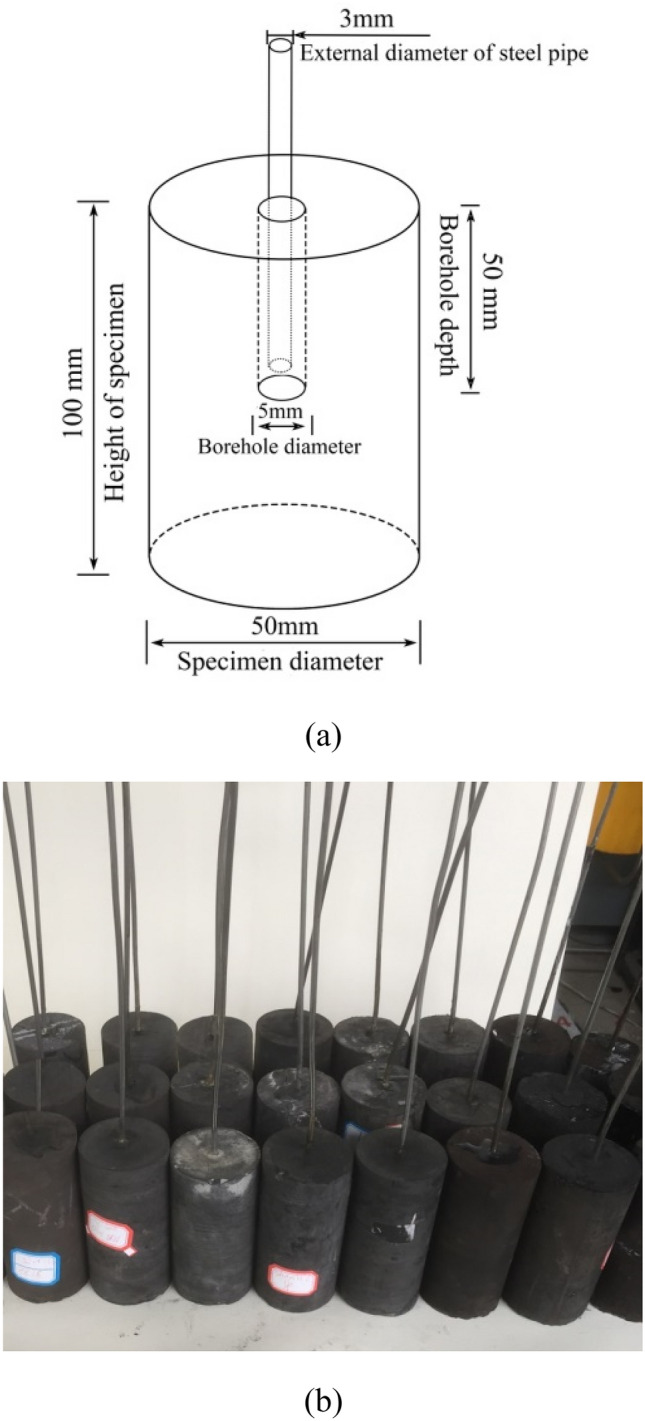
(**a**) Dimensions of specimens used in the fracturing experiments. (**b**) Photograph of the coal and mudstone specimens.

**Figure 4:**
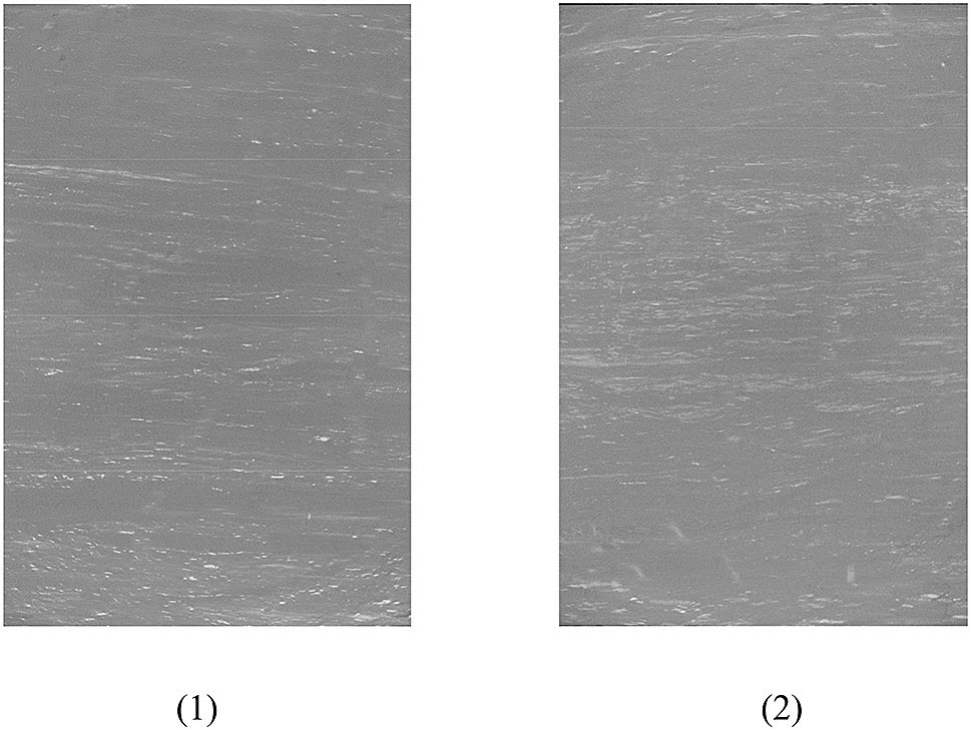
CT scan section image of the borehole for (1) the fat coal specimen and (2) the anthracite specimen.

The specimens used in the fracturing experiments were cylindrical-shaped. A special coring method was employed to obtain the coal and mudstone specimens to reduce mechanical damage on the samples. The core samples were manufactured using a 1.5 mm carborundum wire saw in a computer numerical control machine tool with lubricating oil coolant. As depicted in Fig. 3a, the core samples of diameter 50 mm were trimmed by saw to a length of approximately 100 mm, and then a central borehole of diameter 5 mm was drilled to a depth of 50 mm. Next, a steel pipe for fluid injection (with external and internal diameter of 3 mm and 2 mm, respectively) was inserted into the borehole to simulate the geometry of CBM fracturing wells. The injection pipe was adhered to the preformed borehole using No. 7102 epoxy glue. A photograph of the coal and mudstone fracturing specimens used in the experiments is shown in Fig. 3b. To observe the integrity of the drilled coal specimens, The CT scanning tests were conducted using the μ-FCB CT test system. Figure 4 shows the CT scanned section images of the borehole of the two types of coal specimens. From Fig. 4, there are no microcracks or defects in the prefabricated holes except for the scratches left by drilling, which means that drilling has not caused significant damage to the coal specimen, and it will not affect the subsequent fracturing test results.

### Test apparatus

**Figure 5:**
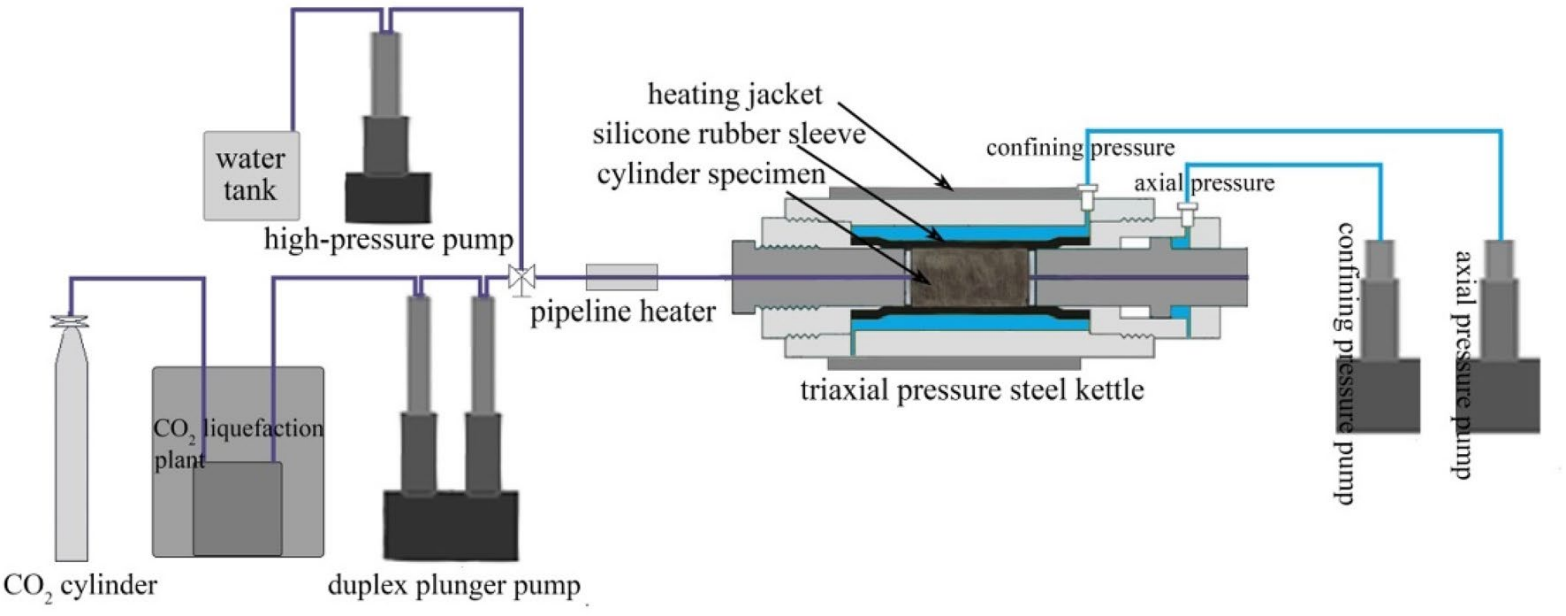
Experimental apparatus for water, L-CO_2_ and Sc-CO_2_ fracturing tests.

The fracturing and permeability experiments were carried out on a triaxial fracturing apparatus designed in-house. As illustrated in Fig. 5, the fracturing apparatus was composed of five subsystems: a triaxial loading and control system, a temperature control system, a water fracturing system, a waterless fracturing system (for L-CO_2_ and Sc-CO_2_ fracturing), and a computer control system. In the triaxial pressure steel kettle, a silicone rubber sleeve was used around the specimen to facilitate the enforcement of the confining pressure. Axial and confining pressure was applied to the cylindrical specimen using axial and confining servo pumps, respectively, with a control precision of 0.02 MPa. The hydraulic fracturing experiments were conducted using a liquid pressure pump. The waterless fracturing system includes a CO_2_ cylinder, a CO_2_ liquefaction plant, a duplex plunger pump (for injecting L-CO_2_) and a counterbalance valve. The temperature control system mainly consisted of a heating jacket for the triaxial pressure steel kettle and pipeline heater, with a temperature control accuracy of 0.1 °C. When performing Sc-CO_2_ fracturing tests, the temperature of the specimen and the pipeline were controlled above the critical temperature (31.10 °C) to ensure that the injected CO_2_ was in the supercritical state. Throughout the fracturing experimental procedure, the axial stress, confining pressure, temperature, and fracturing fluid pressure were monitored and recorded in real time by the computer control system.

### Experiment procedure

For the fracturing tests, the coal core samples were placed into the triaxial pressure steel kettle, and the axial and confining pressures were applied alternately at a rate of 2 MPa min^−1^ to avoid stress damage to the samples. In all the experiments, the axial pressure, *σ*_*a*_, and the confining pressure, *σ*_c_, were set to be 10 MPa and 8 MPa, respectively. For the water fracturing tests, the test temperature was set to 40 °C, and water was injected into the borehole at a constant flow rate of 20 ml min^−1^. The sample was fractured when we observed a rapid drop in the fracturing fluid pressure and a jump in the confining pressure. Prior to the non-aqueous (L-CO_2_ and Sc-CO_2_) fracturing experiments, the CO_2_ gas was liquefied in the CO_2_ liquefaction plant. For the L-CO_2_ fracturing tests, the specimen was not required to be heated, and L-CO_2_ was injected into the borehole at room temperature (21 °C) using a flow rate of 20 ml min^−1^. The physical characteristics of Sc-CO_2_ are very sensitive to temperature variation^36^; therefore, prior to the Sc-CO_2_ fracturing experiments, the gas pipeline and core sample were heated to 40 °C, and the temperature was allowed to stabilize over a period of two hours before injecting Sc-CO_2_. During all the fracturing experiments, a high-precision pressure sensor recorded the fracturing fluid pressure every 0.1 s. Permeability is one of the most important properties required for evaluating the fracturing effects of different fluids; hence, the permeabilities of the specimens were measured using a steady-state method before and after the fracturing experiments. Finally, the surface topographies of the induced fractures were characterized using a 3-D morphological scanner. The exact conditions under which the different tests were carried out are shown in Table 2. For each type of fracturing fluid, experiments were performed on at least two specimens.

**Table 2:** Experimental conditions for the fluid fracturing experiments.

Sample number	Specimen type	Fracturing fluid	Axial, confining pressure (*σ*_*a*_ /*σ*_c_) (MPa)	Temperature (°C)	Fluid injection rate (mL/min)
F1#_water_	Fat coal	Water	10/8	40	20
F2#_water_	Fat coal	Water	10/8	40	20
F1$\#_{\text{L-CO}_{2}}$	Fat coal	L-CO_2_	10/8	21 (room temperature)	20
F2$\#_{\text{L-CO}_{2}}$	Fat coal	L-CO_2_	10/8	21 (room temperature)	20
F1$\#_{\text{Sc-CO}_{2}}$	Fat coal	Sc-CO_2_	10/8	40	20
F2$\#_{\text{Sc-CO}_{2}}$	Fat coal	Sc-CO_2_	10/8	40	20
A1#_water_	Anthracite	Water	10/8	40	20
A2#_water_	Anthracite	Water	10/8	40	20
A1$\#_{\text{L-CO}_{2}}$	Anthracite	L-CO_2_	10/8	21 (room temperature)	20
A2$\#_{\text{L-CO}_{2}}$	Anthracite	L-CO_2_	10/8	21 (room temperature)	20
A1$\#_{\text{Sc-CO}_{2}}$	Anthracite	Sc-CO_2_	10/8	40	20
A2$\#_{\text{Sc-CO}_{2}}$	Anthracite	Sc-CO_2_	10/8	40	20
M1#_water_	Mudstone	Water	10/8	40	20
M2#_water_	Mudstone	Water	10/8	40	20
A1$\#_{\text{L-CO}_{2}}$	Mudstone	L-CO_2_	10/8	21 (room temperature)	20
A2$\#_{\text{L-CO}_{2}}$	Mudstone	L-CO_2_	10/8	21 (room temperature)	20
A1$\#_{\text{Sc-CO}_{2}}$	Mudstone	Sc-CO_2_	10/8	40	20
A2$\#_{\text{Sc-CO}_{2}}$	Mudstone	Sc-CO_2_	10/8	40	20

## Results and discussion

### Breakdown pressures for the different fracturing fluids

**Figure 6:**
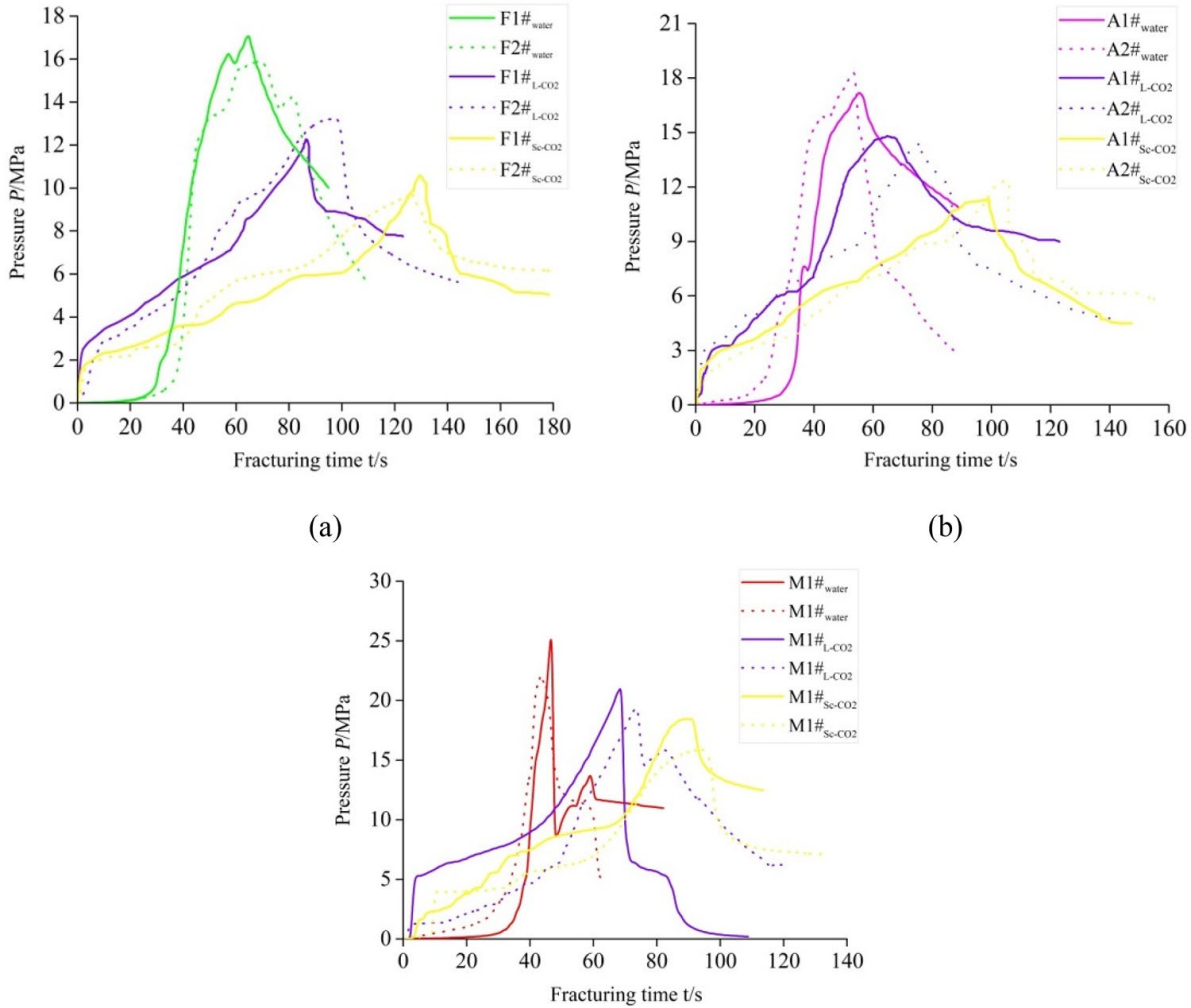
Comparison of fracturing test results with different fracturing fluids for the (**a**) fat coal, (**b**) anthracite, and **(c**) mudstone specimens for different fracturing fluids.

The fracturing fluid pressure versus time curves for the fat coal, anthracite and mudstone samples are plotted in Fig. 6 for all three kinds fracturing fluids. As the figure shows, for all the three sample types, the hydraulic pressure increased slowly in the initial stages of water fracturing. When the water is injected, the fluid pressure increased rapidly until fracture occurred. In comparison, for the waterless fracturing experiments, the increase rate of fluid pressure become significantly slower after reaching a certain value and thus it takes longer than hydraulic fracturing to reach the breakdown pressure. A possible explanation is that at the onset of waterless fracturing, the large diffusion coefficients of L-CO_2_ and Sc-CO_2_ enable them to quickly fill up the borehole of the tested specimens, which results in a rapid increase of fluid pressure, but after that, the fluid pressure accumulated slowly due to the high compressibility of CO_2_.

The experimental results of fracturing with all the three fluids are presented in Table 3 for the fat coal, anthracite, and mudstone samples. As the table shows, the breakdown pressures of waterless fracturing were remarkably lower than that of water fracturing. Among the three fracturing fluids, the breakdown pressures in Sc-CO_2_ fracturing were the lowest, which are 16.65%, 12.25% and 11.83% lower than L-CO_2_ for the fat coal, anthracite, and mudstone samples, respectively, and 38.25%, 30.93% and 25.51% lower than hydraulic fracturing for the same three sample types, respectively. Moreover, the high-rank coal exhibited consistently higher breakdown pressures than the mid-rank coal. According to the classical failure criteria in hydraulic fracturing^37^, when the tangential stress near the fracturing wellbore reaches the tensile strength of the coal or rock, the fracture forms and the corresponding fluid pressure in the wellbore is referred to as the breakdown pressure. The critical tangential stress includes the geological stress *σ*_θ_^1^ resulting from the two principal stresses *σ*_*a*_ and *σ*_c_, and the fluid stress *σ*_θ_^2^ induced by the fracturing fluid pressure, *P*, in the wellbore. The equation for the stress near the fracturing wellbore is expressed as follows:

1 $$\mathop \sigma \nolimits_{{_{\uptheta } }}^{{1}} { + }\mathop \sigma \nolimits_{{_{\uptheta } }}^{{2}} { = }\mathop \sigma \nolimits_{{t}} {\kern 1pt} {\kern 1pt} {\kern 1pt} {\kern 1pt} {\kern 1pt} {\kern 1pt} {\kern 1pt} {\kern 1pt} {\kern 1pt} {(at}{\kern 1pt} {\kern 1pt} {\kern 1pt} r{ = R)}$$

where *r* and* θ* are the cylindrical coordinates, *R* is the radial distance away from the fracturing wellbore at which the rock fractures, and *σ*_t_ is the rock tensile strength. The individual stress expressions for *σ*_θ_^1^ and *σ*_θ_^2^ are

**Table 3:** Fracturing test results for different fracturing fluids in the (a) fat coal (F*x*#), (b) anthracite (A*x*#) and (c) mudstone (M*x*#) specimens.

Sample number	Fracturing fluid	Breakdown pressure (MPa)		Reduction rate (%)	Initiation time (s)		Increase rate (%)
		Test value	Average value		Test value	Average value	
**(a)**							
F1#_water_	Water	16.27	16.76	0	68.31	66.73	0
F2#_water_	Water	17.25			65.14		
F1$\#_{\text{L-CO}_{2}}$	L-CO_2_	13.25	13.14	− 21.60	98.15	96.26	+ 44.25%
F2$\#_{\text{L-CO}_{2}}$	L-CO_2_	13.03			94.37		
F1$\#_{\text{Sc-CO}_{2}}$	Sc-CO_2_	10.88	10.35	− 38.25	127.12	129.22	+ 93.65%
F2$\#_{\text{Sc-CO}_{2}}$	Sc-CO_2_	9.82			131.32		
**(b)**							
A1#_water_	Water	17.25	17.88	0	55.28	54.87	0
A2#_water_	Water	18.51			54.46		
A1$\#_{\text{L-CO}_{2}}$	L-CO_2_	14.95	14.54	− 18.68	78.15	80.70	+ 47.07%
A2$\#_{\text{L-CO}_{2}}$	L-CO_2_	14.13			83.24		
A1$\#_{\text{Sc-CO}_{2}}$	Sc-CO_2_	12.89	12.35	− 30.93	114.27	111.18	+ 102.62%
A2$\#_{\text{Sc-CO}_{2}}$	Sc-CO_2_	11.81			108.09		
**(c)**							
M1#_water_	Water	25.14	23.83	0	47.59	45.71	0
M2#_water_	Water	22.52			43.82		
M1$\#_{\text{L-CO}_{2}}$	L-CO_2_	21.38	20.57	− 13.68	69.23	71.71	+ 56.88%
M2#_L-CO2_	L-CO_2_	19.76			74.18		
M1$\#_{\text{Sc-CO}_{2}}$	Sc-CO_2_	16.82	17.75	− 25.51	91.25	89.54	+ 95.89%
M2$\#_{\text{Sc-CO}_{2}}$	Sc-CO_2_	18.68			87.82		

:

2 $$\mathop \sigma \nolimits_{{_{\theta } }}^{{1}} = \frac{{\sigma _{a} + \sigma _{c} }}{2}\left( {1 + \frac{{R^{2} }}{{r^{2} }}} \right) - \frac{{\sigma _{a} - \sigma _{c} }}{2}\left( {1 + 3\frac{{R^{2} }}{{r^{2} }}} \right)\cos \left( {{2}\theta } \right)$$

3 $$\mathop \sigma \nolimits_{{_{\theta } }}^{{2}} = \frac{{R^{2} }}{{r^{2} }}P$$

When *P* arrives at the breakdown pressure, *P*_b_, the tangential stress near the fracturing wellbore reaches *σ*_t_ and the rock begins to break. Substituting Eqs. (2) and (3) into Eq. (1) allows *P*_b_ to be expressed as follows:

4 $$P_{b} = \sigma_{t} - (3\sigma_{c} - \sigma_{a} )$$

Equation (4) applies to aqueous fracturing, neglecting the pore pressure variation of the target rock strata near the wellbore. This is because it is difficult for the aqueous medium to permeate into the pores of the rock under high stress conditions. Hence the effect of the pore water pressure on the breakdown pressure is not considered in classical hydraulic fracturing models. However, compared to the water, Sc-CO_2_ and L-CO_2_ have lower viscosity and surface tension, which facilitates penetration into the coal pores^38^. The Sc-CO_2_ or L-CO_2_ penetration causes additional tangential pore stress, *σ*_θ_^3^, around the fracturing wellbore^39^:

5 $$\sigma ^{3} _{\theta } = \frac{{\alpha (1 - 2\nu )}}{{1 - \nu }}\left[ {\frac{1}{{r^{2} }}\int_{R}^{r} {p(r)rdr - p(r)} } \right]$$

where *ν* is the Poisson’s ratio of the rock, *α* is the Biot coefficient, defined as $\alpha =1-\frac{{K}_{b}}{{K}_{S}}$, where *K*_b_ and *K*_s_ are the elasticity moduli of the bulk rock and the rock matrix, respectively, and *p*(*r*) is the pore pressure at a distance* r* from the fracturing wellbore. Based on Terzaghi’s effective stress principle^40^, *σ*_θ_^3^ may be incorporated into Eq. (1) as follows:

6 $$\mathop \sigma \nolimits_{{_{\theta } }}^{{1}} + \mathop \sigma \nolimits_{{_{\theta } }}^{{2}} + \mathop \sigma \nolimits_{{_{\theta } }}^{3} = \mathop \sigma \nolimits_{t} - p{\kern 1pt} {\kern 1pt} {\kern 1pt} {\kern 1pt} {\kern 1pt} {\kern 1pt} {\kern 1pt} {\kern 1pt} {\kern 1pt} (at{\kern 1pt} {\kern 1pt} {\kern 1pt} r = R)$$

According to Eq. (5), *p* is equal to *P* at the fracturing wellbore wall, i.e., *r* = *R*. Therefore, *σ*_θ_^3^ becomes $-P\alpha (1-2\nu )/( 1-\nu )$. Substituting this expression for *σ*_θ_^3^ into Eq. (6), the following expression for the breakdown pressure is obtained:

7 $$P_{b} = \frac{{\sigma_{t} - (3\sigma_{c} - \sigma_{a} )}}{{2 - \alpha \frac{1 - 2\nu }{{1 - \nu }}}}{\kern 1pt} {\kern 1pt} {\kern 1pt}$$

Equation (7) allows the breakdown pressures of waterless fracturing to be calculated. Compared to Eq. (4) for water fracturing, Eq. (7) contains the extra multiplier factor $1/(2-\alpha (1-2\nu )/(1-\nu ))$. Equation (7) shows that for rocks, *ν* varies between 0 and 0.5, and *α* is always less than 1 for rocks. Therefore, the factor $1/(2-\alpha (1-2\nu )/(1-\nu ))$ ranges between 0.5 and 1. This explains why the breakdown pressures driven by L-CO_2_ and Sc-CO_2_ are lower than that induced by water.

### Crack propagation path and fracture surface topography

**Figure 7:**
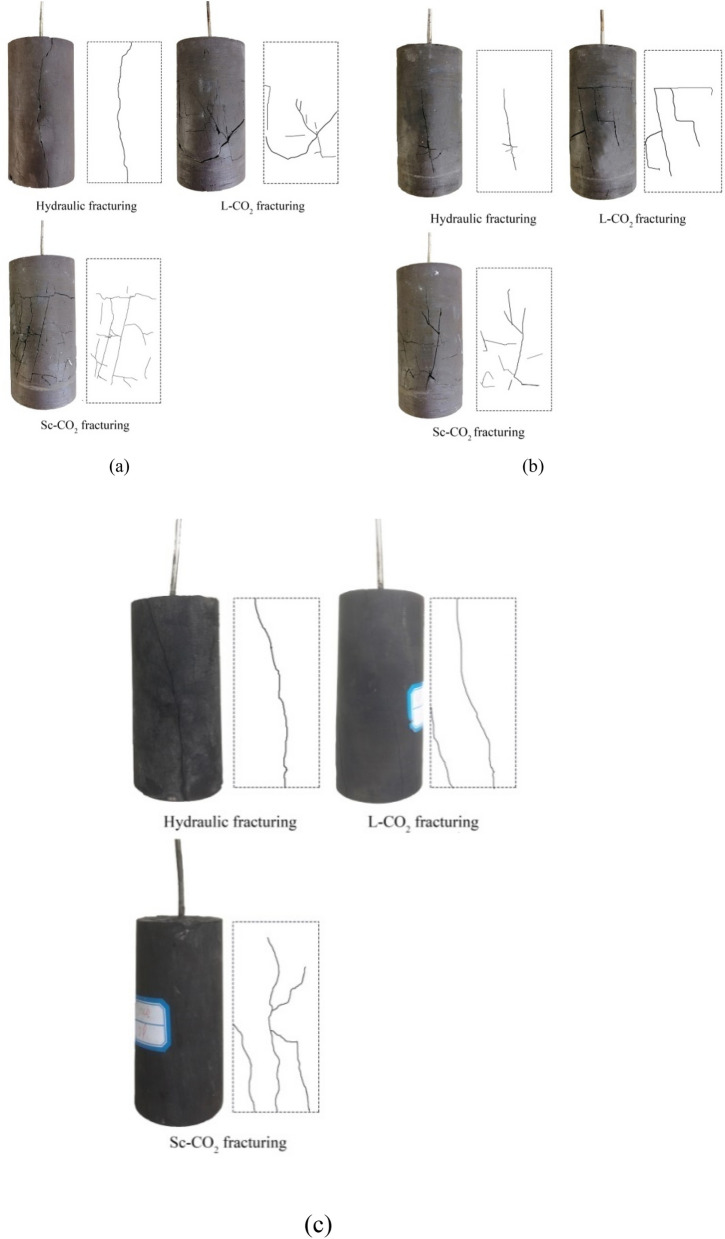
Crack propagation paths for the (**a**) fat coal, (**b**) anthracite, and (**c**) mudstone samples fractured by water, L-CO_2_ and Sc-CO_2_.

Figure 7 compares the crack propagation paths in the three types of samples fractured by each fluid, with sketches of the induced fractures shown alongside photographs of the cylindrical samples for ease of comparison. As the figure shows, for water fracturing, macroscopic through-crack propagation occurred in the samples, whereas several secondary cracks were induced after L-CO_2_ fracturing. For Sc-CO_2_ fracturing, the number of induced cracks was much more than for the other two fracturing fluids, with a wide and complex fracture network appearing in the fractured specimens.

**Figure 8:**
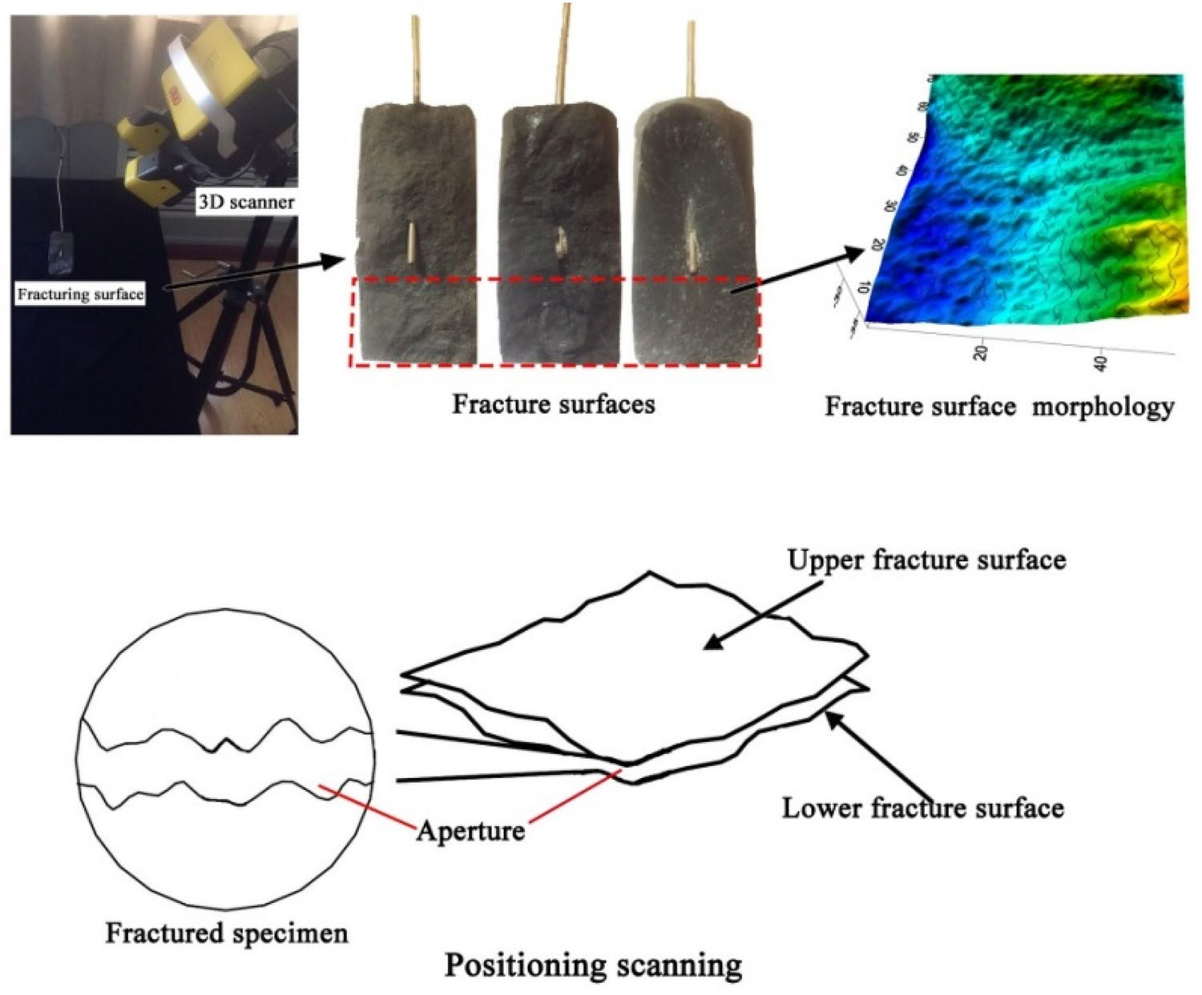
Fracture surface morphology and positional scanning^5^.

Measurements of fracture surface morphology were also carried out (Fig. 8). As the figure shows, the fractured specimens were split into two halves by the main crack, and the 3D scanner was used to measure the morphologies of the fractured surfaces in the portions without the fracturing boreholes, thereby mapping the three-dimensional coordinates of the induced fractures. This enabled the areas of the fracture surfaces to be calculated by multi-faceted function fitting of the morphology points. The three-dimensional roughness coefficient, *R*_s_, of the fracture surface induced by the different fluids is defined as follows:

8 $$R_{{s}} = \frac{{A_{{t}} }}{{A_{{n}} }}$$

where *A*_t_ is the three-dimensional area of the fracture surface and *A*_n_ is the projection area of the fracture surface. The larger the value of *R*_s_, the more tortuous the crack surface. The *R*_s_ values for the scanned specimens are listed in Table 4. As the table shows, for all three sample types the *R*_s_ values of the fractures induced by Sc-CO_2_ were largest and that by water fracturing were the lowest. Hence, the fracture surfaces induced by Sc-CO_2_ are rougher and more tortuous. Among the three sample types, the *R*_s_ values of the fat coal were larger than those of the anthracite for all the three fracturing fluids, and the *R*_s_ values of mudstones are the lowest, which indicates that the lower the coal rank, the more tortuous the crack surface.

**Table 4:** Roughness coefficients and the average fracture apertures for different fracturing fluids in the (a) fat coal (F*x*#), (b) anthracite (A*x*#) and mudstone (M*x*#) specimens.

Sample number	Fracturing fluid	Roughness coefficients *R*_*S*_		Average fracture aperture *e*_m_ (mm)	
		Test value	Average value	Test value	Average value
**(a)**					
F1#_water_	Water	1.217	1.256	0.692	0.732
F2#_water_	Water	1.295		0.772	
F1$\#_{\text{L-CO}_{2}}$	L-CO_2_	1.256	1.317	0.575	0.618
F2$\#_{\text{L-CO}_{2}}$	L-CO_2_	1.378		0.661	
F1$\#_{\text{Sc-CO}_{2}}$	Sc-CO_2_	1.433	1.506	0.401	0.426
F2$\#_{\text{Sc-CO}_{2}}$	Sc-CO_2_	1.579		0.451	
**(b)**					
A1#_water_	Water	1.182	1.215	0.593	0.625
A2#_water_	Water	1.248		0.657	
A1$\#_{\text{L-CO}_{2}}$	L-CO_2_	1.203	1.276	0.488	0.513
A2$\#_{\text{L-CO}_{2}}$	L-CO_2_	1.349		0.538	
A1$\#_{\text{Sc-CO}_{2}}$	Sc-CO_2_	1.391	1.423	0.378	0.406
A2$\#_{\text{Sc-CO}_{2}}$	Sc-CO_2_	1.455		0.434	
**(c)**					
M1#_water_	Water	1.051	1.186	0.418	0.452
M2#_water_	Water	1.321		0.486	
M1$\#_{\text{L-CO}_{2}}$	L-CO_2_	1.107	1.205	0.317	0.368
M2$\#_{\text{L-CO}_{2}}$	L-CO_2_	1.303		0.419	
M1$\#_{\text{Sc-CO}_{2}}$	Sc-CO_2_	1.258	1.321	0.213	0.255
M2$\#_{\text{Sc-CO}_{2}}$	Sc-CO_2_	1.284		0.297	

Further, the fracture apertures of the specimens under each experimental condition were determined by positional scanning, using the point-cloud data matching method proposed by Chen et al*.*^41,42^. This process is illustrated in Fig. 8. First, the spatial coordinates of the exterior surface of the fractured sample were captured by the 3D scanner (whose precision ranges from 0.01–0.02 mm) and selected as the reference position; next, the sample was opened up and both the fracture surfaces were scanned. By matching the reference position and coordinates of the two surfaces, the fracture aperture can be calculated based on the distances of each grid point:

9 $$e_{i} = z_{i}^{ + } - z_{i}^{ - }$$

10 $$e_{{m}} = \frac{{\sum\nolimits_{i = 1}^{N} {e_{i} } }}{N}$$

where *e*_i_ is the vertical distance of each grid point, $z_{i}^{ + }$ is *z*-elevation of the top fracture surface and $z_{i}^{ - }$ is *z*-elevation of the bottom fracture surface; *e*_m_ the average fracture aperture, and *N* the total number of grid points of the fracture surface. As shown in Table 4, the average single fracture apertures of the water-induced cracks were the largest among the different fracturing fluids for all three sample types. Compared to the high-rank anthracite coal, the fat coal specimens had wider fracture apertures regardless of fracturing fluid.

The viscosity of the fracturing fluid is one of the most important factors influencing the crack propagation path and fracture surface topography. The fluid with low viscosity is easier to permeate into the micro-pores, and as such leads to the longer crack length and rougher fracture surfaces. Under temperature and pressure conditions of 15 MPa and 40 °C, respectively, the viscosity of Sc-CO_2_ (69.56 μPas) is much less than those of L-CO_2_ and water (621.56 μPas and 903.62 μPas, respectively)^43,44^. According to Philibert^45^, the diffusion range of fracturing fluids in rocks may be represented by the average diffusion length, *x*_*d*_, from Einstein’s diffusion equation:

11 $$x_{d} = \sqrt {4D_{d} \cdot t}$$

where *D*_*d*_ is the diffusion coefficient of the fracturing fluid, whose values are 2.71 × 10^−8^ m^2^/s, 1.08 × 10^−8^ m^2^/s, and 5.32 × 10^−9^ m^2^/s for Sc-CO_2_, L-CO_2_, and H_2_O, respectively, and *t* is the diffusion time. The *x*_*d*_ values calculated using Eq. (11) for the three fluids (with the average times taken for fracture initiation following fluid injection in each sample type used as the *t* values) were 3453.11 μm, 1916.25 μm, and 1081.85 μm for Sc-CO_2_, L-CO_2_ and water, respectively. Therefore, the diffusion range of Sc-CO_2_ in the samples was much larger than that of water. This is consistent with the observation from Fig. 7, which shows that the Sc-CO_2_ caused more widely distributed cracks.

The capillary effect is another important factor determining the propagation of induced cracks, as it affects the intrusion of fracturing fluid into the coal matrix. The fracturing fluids can only enter the pores of coal specimens once the pore diameter exceeds the critical pore diameter, d^*^, which is defined as follows:

12 $$d^{*} = \frac{{4\sigma_{i} \cos \theta }}{{P_{c}^{*} }}$$

where *σ*_*i*_ is the interfacial tension of the fracturing fluid in coal, *θ* is the contact angle, *P*_c_^*^ is the entry pressure. In coals, the entry pressure *P*_c_^*^ is typically ~ 10 MPa, and the interfacial tensions of Sc-CO_2_, L-CO_2_, and water are 25 mN/m, 45 mN/m, and 55 mN/m^46^. In addition, the contact angle of Sc-CO_2_ and L-CO_2_ is ~ 0°, while that of water is ~ 16°^47^. From Eq. (12), the threshold diameter of pores for the permeation of Sc-CO_2_ is 100 nm, whereas that for water is 211 nm. This suggests that Sc-CO_2_ is capable of entering the coal matrix through smaller pores, which may explain why Sc-CO_2_ fracturing leads to more extensive fractured areas.

**Figure 9:**
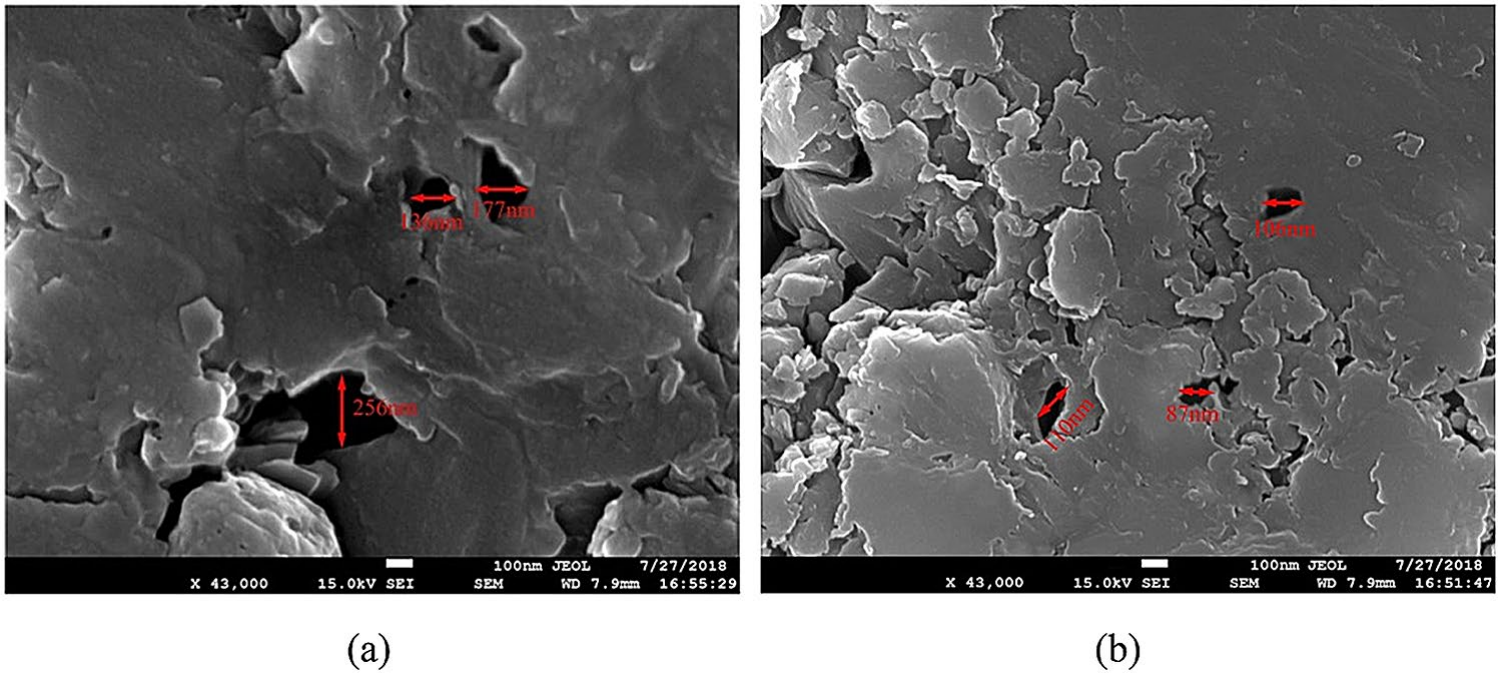
Micro-pore structures of the (**a**) fat coal and (**b**) anthracite specimens.

Figure 9 shows scanning electron microscopy (SEM) images of the pore structures of the fat coal and anthracite samples at 43,000× magnification, obtained using a JIB-4700F (JEOL, Japan) instrument. During the SEM analysis, the coal samples were viewed under 15 kV voltage with a spot size of 4.5 under low vacuum mode. As the figure shows, the pore diameters of the two kinds of coals are generally between 100 and 200 nm (the pore diameter of the fat coal sample is about 100 nm, and the pore diameter of the anthracite sample is about 200 nm). Hence, Sc-CO_2_ can enter the coal through the pores which can cause more extensive cracking, whereas it is more difficult for water to get into pore structure. Moreover, the pore diameters of the fat coal appear to be larger than those of the anthracite, which may explain the fact that the cracks induced by Sc-CO_2_ in the former were more extensive than those in the latter (see Fig. 7).

### Permeability of the fractured coal specimens

After the fracturing tests, the permeabilities of the fractured specimens were measured by steady-state flow methods^48,49^ under the same axial pressure as in the fracturing tests and the confining pressures were set to 4, 6, 8, 10 and 12 MPa. Nitrogen was used for the permeability measurements at a seepage pressure of 2 MPa to avoid absorption effects. Before the formal seepage experiment, Nitrogen is continuously injected into the fractured coal specimen for an hour to remove the fracturing fluid remaining in the fracture. The nitrogen flow in the fractures may be estimated using the following cubic equation^50^:

13 $$q = - \frac{{e^{3} }}{12\mu }\frac{\partial P}{{\partial x}}$$

where *e* is the fracture aperture, *μ* is the fluid viscosity coefficient, and $\partial P/\partial x$ is the pressure gradient of the fluid. Witherspoon et al*.*^51^ took into account the impact of the fracture roughness to modify Eq. (13), as follows:

14 $$q = - \frac{{e^{3} }}{12\mu }\frac{\partial P}{{\partial x}}\frac{1}{{1 + 6(\frac{\Delta }{{\overline{e}}})^{1.5} }}$$

where $\stackrel{-}{e}$ is the average effective hydraulic fracture aperture, and *Δ* is the average height of the coordinate point measured by the 3D scanner. The permeabilities of the cylindrical specimens both before and after the fracturing tests are given by Darcy’s law:

15 $$q = - \left( {\frac{{k{{\cdot}}A}}{\mu }{\text{ + }}\frac{1}{{W_{f} }}} \right)\frac{{\partial P}}{{\partial x}}$$

where *A* is cross-sectional area of sample along the seepage direction and *k* is the permeability; 1/*W*_*f*_ is the fluid compressibility. Substituting Eq. (14) into Eq. (15), the relationship between $\stackrel{-}{e}$ and *k* is established as:

16 $$\frac{{\overline{e}^{3} }}{{12{ + }72\left( {\frac{\Delta }{{\overline{e}}}} \right)^{1.5} }}{ = }k \cdot A$$

**Figure 10:**
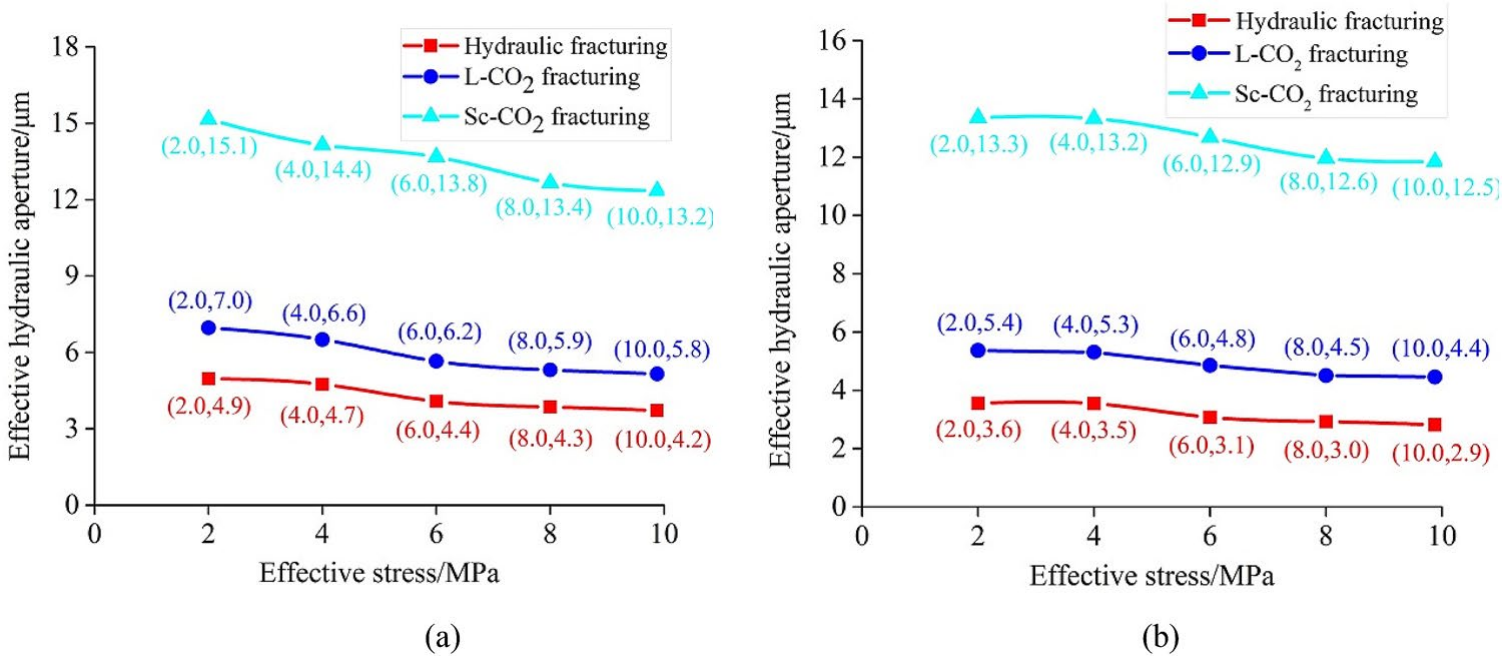
The effective hydraulic aperture of the fractured (**a**) fat coal and (**b**) anthracite specimens after fracturing with different fluids.

The $\stackrel{-}{e}$ values for the different specimens calculated using Eq. (16) are plotted in Fig. 10. As the figure shows, for the fat coal specimens, the value of $\stackrel{-}{e}$ after Sc-CO_2_ fracturing was 3.05 times greater than that after hydraulic fracturing and 2.18 times greater than that after L-CO_2_ fracturing. Similarly, for the anthracite specimens, the value of $\stackrel{-}{e}$ after Sc-CO_2_ fracturing was 3.75 and 2.49 times greater than after hydraulic and L-CO_2_ fracturing, respectively. This means that Sc-CO_2_ can greatly increase the permeability of coals compared to the other two fluids, which is beneficial to CBM recovery. There are two possible mechanisms that can explain the greater effective hydraulic apertures induced by Sc-CO_2_ compared to the other two fluids. Firstly, Sc-CO_2_ fracturing may release more energy which produces more cracks in coal specimens, resulting in substantial increase in permeability. Secondly, Sc-CO_2_ can seepage through smaller pore than the other two fluids, causing greater damage and micro-cracking to the solid matrix. This second mechanism works as follows: during the fracturing and crack propagation processes, Sc-CO_2_ enters small pores and weakens the mineral cementation; as a result, mineral grains may be peeled off from the fracture surface and remain in the induced fractures, thus keeps the cracks open against closure stresses. Moreover, the $\stackrel{-}{e}$ values of the fat coal specimens were greater than those of anthracite specimens, and showed a steady decrease with increasing effective stress (Fig. 10).

## Conclusions

In this study, fracturing experiments were conducted with two different kinds of coal (fat coal and anthracite) and one type of mudstone. Three different fracturing fluids are adopted, including water, liquid CO_2_ (L-CO_2_), and supercritical CO_2_ (Sc-CO_2_). The fracture surfaces induced by the different fluids were 3D scanned to quantify the roughness and the fracture apertures of the specimens. Furthermore, the permeabilities of the specimens were measured after fracturing. The main conclusions from this study are summarized as follows:

1. The breakdown pressures of waterless fracturing in the coal specimens were significantly lower than that of hydraulic fracturing. Among them, the breakdown pressures of Sc-CO_2_ fracturing is the lowest, 16.65%, 12.25%, and 11.83% lower than L-CO_2_ fracturing and 38.25%, 30.93% and 25.51% lower than water hydraulic fracturing for the fat coal, anthracite, and mudstone specimens, respectively. Moreover, the high-rank anthracite specimens exhibited higher breakdown pressures than the mid-rank coal fat coal specimens for all three fracturing fluids.
2. Sc-CO_2_ fracturing induced far more cracks than L-CO_2_, and formed wide and complex fracture networks. Measurements of fracture surface morphology were carried out, which showed that the three-dimensional roughness coefficient, *R*_s_, of the fractures induced by Sc-CO_2_ were larger than those caused by the other two fracturing fluids for all three samples types. Therefore, the fracture surfaces created by Sc-CO_2_ are rougher and more tortuous. Meanwhile, the *R*_s_ values for the fat coal were greater than those for the anthracite regardless of fracturing fluid, which indicates that more tortuous crack surfaces may take place in lower rank coals. Moreover, the fracture apertures of the specimens were determined by positioning scanning, whose results shows that the average single fracture apertures of the ScCO_2_-induced cracks were the smallest amongst the three fracturing fluids.
3. Viscosity of the fracturing fluid is the key factors influencing the crack propagation path and fracture surface topography. The diffusion lengths of Sc-CO_2_, L-CO_2_, and water were estimated to be 3453.11 μm, 1916.25 μm, and 1081.85 μm, respectively; hence, the diffusion range of Sc-CO_2_ in the specimens is larger than the other two fluids. The capillary effect is another critical factor that influences the propagation of induced cracks, as it has a bearing on the intrusion of fracturing fluid into the coal matrix. The threshold pore diameters that allow the permeation of Sc-CO_2_ and water into the coal body were estimated to be 100 nm and 211 nm, respectively, suggesting that Sc-CO_2_ is more likely than water to permeate into the coal matrix through smaller pores during the fracturing fluid injection. Hence, the more extensive fractured area as seen in the specimens injected with Sc-CO_2_ may be attributed to these two factors (i.e. diffusion length and capillary effects).
4. For the fat coal specimens, the effective hydraulic aperture, $\stackrel{-}{e}$, caused by Sc-CO_2_ fracturing was 3.05 times larger than that caused by water fracturing and 2.18 times greater than that induced by L-CO_2_ fracturing. Similarly, for the anthracite specimens, the $\stackrel{-}{e}$ value caused by Sc-CO_2_ fracturing was 3.75 and 2.49 times greater than water and L-CO_2_ fracturing, respectively. These results demonstrate that Sc-CO_2_ fracturing considerably improves the permeability of coals and is therefore beneficial to CBM recovery.

## References

[1] Mastalerz M, Drobniak A (2020). Coalbed methane: Reserves, production, and future outlook. Future Energy.

[2] Karacan CÖ, Ruiz FA, Cotè M, Phipps S (2011). Coal mine methane: A review of capture and utilization practices with benefits to mining safety and to greenhouse gas reduction. Int. J. Coal Geol..

[3] Liu CL, Jie Z, Cai CB, Yang HL, Fan MZ (2009). Methodologies and results of the latest assessment of coalbed methane resources in China. Nat. Gas. Ind..

[4] Tao S, Pan Z, Tang S, Chen S (2019). Current status and geological conditions for the applicability of cbm drilling technologies in china: A review. Int. J. Coal Geol.

[5] Yang, J., Liang, W., Lian, H., Chen, Y. & Li, L. Influence of the Different Fluids on the Hydraulic Fracturing Mechanism of Sandy Mudstone. In *ISRM International Symposium-10th Asian Rock Mechanics Symposium*. International Society for Rock Mechanics and Rock Engineering, January (2018).

[6] Palmer I (2010). Coalbed methane completions: A world view. Int. J. Coal Geol..

[7] Holditch, S. A., Ely, J. W., Semmelbeck, M. E., Carter, R. H., Hinkel, J. & Jeffrey, Jr R. G. Enhanced recovery of coalbed methane through hydraulic fracturing. In *SPE annual technical conference and exhibition*. Society of Petroleum Engineers, January (1988).

[8] Meng Y, Tangm D, Xu H, Li Y, Gao L (2014). Coalbed methane produced water in China: Status and environmental issues. Environ Sci. Pollut. Res..

[9] Cooley H, Donnelly K, Ross N, Luu P (2012). Hydraulic Fracturing and Water Resources: Separating the Frack from the Fiction.

[10] Middleton RS, Carey JW, Currier RP, Hyman JD, Kang Q, Karra S, Viswanathan HS (2015). Shale gas and non-aqueous fracturing fluids: Opportunities and challenges for supercritical CO_2_. Appl. Energy..

[11] Middleton R, Viswanathan H, Currier R, Gupta R (2014). CO_2_ as a fracturing fluid: Potential for commercial-scale shale gas production and CO_2_ sequestration. Energy Proced..

[12] Liu H, Wang F, Zhang J, Meng S, Duan Y (2014). Fracturing with carbon dioxide: Application status and development trend. Pet. Explor. Dev..

[13] Han Y, Zheng H, Jing X, Zheng L (2018). Swelling behavior of polyester in supercritical carbon dioxide. J. CO2 Util..

[14] Perera MSA, Ranjith PG, Airey DW, Choi SK (2011). Sub- and super-critical carbon dioxide flow behavior in naturally fractured black coal: An experimental study. Fuel.

[15] Siemons N, Busch A (2007). Measurement and interpretation of supercritical CO_2_ sorption on various coals. Int. J. Coal Geol..

[16] Yu H, Yuan J, Guo W, Cheng J, Hu Q (2008). A preliminary laboratory experiment on coalbed methane displacement with carbon dioxide injection. Int. J. Coal Geol..

[17] Gale J, Freund P (2001). Coal-bed methane enhancement with CO_2_ sequestration worldwide potential. Environ. Geosci..

[18] Pei P, Ling K, He J, Liu Z (2015). Shale gas reservoir treatment by a CO_2_-based technology. J. Nat. Gas. Sci. Eng..

[19] Feng G, Kang Y, Sun ZD, Wang XC, Hu YQ (2019). Effects of supercritical CO_2_ adsorption on the mechanical characteristics and failure mechanisms of shale. Energy..

[20] Verdon JJP, Kendall JM, Maxwell SSC (2010). A comparison of passive seismic monitoring of fracture stimulation from water and CO_2_ injection. Geophysics.

[21] Ishida T, Aoyagim K, Niwa T, Chen Y, Murata S, Chen Q, Nakayama Y (2012). Acoustic emission monitoring of hydraulic fracturing laboratory experiment with supercritical and liquid CO_2_. Geophys Res. Lett..

[22] Zhou J, Liu G, Jiang Y, Xian X, Liu Q, Zhang Q, Tan J (2016). Supercritical carbon dioxide fracturing in shale and the coupled effects on the permeability of fractured shale: An experimental study. J. Nat. Gas. Sci. Eng..

[23] Zhang X, Lu Y, Tang J, Zhou Z, Liao Y (2017). Experimental study on fracture initiation and propagation in shale using supercritical carbon dioxide fracturing. Fuel.

[24] Jia Y, Lu Y, Elsworth D, Fang Y, Tang J (2018). Surface characteristics and permeability enhancement of shale fractures due to water and supercritical carbon dioxide fracturing. J. Pet. Sci. Eng..

[25] Zhou D, Zhang G, Wang Y, Xing Y (2018). Experimental investigation on fracture propagation modes in supercritical carbon dioxide fracturing using acoustic emission monitoring. Int. J. Rock Mech. Min. Sci..

[26] Cai C, Kang Y, Wang X, Hu Y, Chen H, Yuan X, Cai Y (2018). Mechanism of supercritical carbon dioxide (SC-CO_2_) hydro-jet fracturing. J. CO2 Util..

[27] Yang J, Liang W, Chen Y, Li L, Lian H (2017). Experiment research on the fracturing characteristics of mudstone with different degrees of water damage. Chin. J. Rock Mech. Eng..

[28] Zhang Y, Zhang Z, Sarmadivaleh M, Lebedev M, Barifcanim A, Yu H, Iglauer S (2017). Micro-scale fracturing mechanisms in coal induced by adsorption of supercritical CO_2_. Int. J. Coal. Geol..

[29] Yang J, Lian H, Liang W, Nguyen VP, Chen Y (2018). Experimental investigation of the effects of supercritical carbon dioxide on fracture toughness of bituminous coals. Int. J. Rock Mech. Min. Sci..

[30] Yang J, Lian H, Liang W, Nguyen VP, Bordas SPA (2019). Model I cohesive zone models of different rank coals. Int. J. Rock Mech. Min. Sci..

[31] Wang J, Elsworth D, Wu Y, Liu J, Zhu W, Liu Y (2018). The influence of fracturing fluids on fracturing processes: A comparison between water, oil and SC-CO_2_. Rock Mech. Rock Eng..

[32] Song W, Ni H, Wang R, Sun B, Shen Z (2017). Pressure transmission in the tubing of supercritical carbon dioxide fracturing. J. CO2 Util..

[33] Yang ZZ, Yi LP, Li XG, Chen YT, Sun J (2018). Model for calculating the wellbore temperature and pressure during supercritical carbon dioxide fracturing in a coalbed methane well. J. CO2 Util..

[34] O'Keefe JM, Bechtel A, Christanis K, Dai S, DiMichele WA, Eble CF, Wagner NJ (2013). On the fundamental difference between coal rank and coal type. Int. J. Coal. Geol..

[35] Li H, Lau HC, Huang S (2018). China’s coalbed methane development: A review of the challenges and opportunities in subsurface and surface engineering. J. Pet. Sci. Eng..

[36] Liao SM, Zhao TS (2002). An experimental investigation of convection heat transfer to supercritical carbon dioxide in miniature tubes. Int. J. Heat. Mass. Transf..

[37] Hubbert MK, Willis DG (1972). Mechanics of hydraulic fracturing. Soc. Pet. Eng. AIME..

[38] Vishal V (2017). In-situ disposal of CO_2_: Liquid and supercritical CO_2_ permeability in coal at multiple down-hole stress conditions. J. CO2 Util..

[39] Ito T (2008). Effect of pore pressure gradient on fracture initiation in fluid saturated porous media. Rock Eng. Fract. Mech..

[40] Ghabezloo S, Sulem J, Guédon S, Martineau F (2009). Effective stress law for the permeability of a limestone. Int. J. Rock Mech. Min. Sci..

[41] Chen Y, Liang W, Lian H, Yang J, Nguyen VP (2017). Experimental study on the effect of fracture geometric characteristics on the permeability in deformable rough-walled fractures. Int. J. Rock Mech. Min. Sci..

[42] Chen Y, Lian H, Liang W, Yang J, Nguyen VP, Bordas SPA (2019). The influence of fracture geometry variation on non-Darcy flow in fractures under confining stresses. Int. J. Rock Mech. Min. Sci..

[43] Kestin J, Sengers JV, Kamgar PB, Sengers JMHL (1984). Thermophysical properties of fluid H_2_O. J. Phys. Chem. Ref. Data..

[44] Phillips P (2006). The viscosity of carbon dioxide. Trans. Proc. R. Soc. A Math. Phys. Eng. Sci..

[45] Gaffney C (2017). One and a half century of diffusion: Fick, Einstein, before and beyond. Diffus. Fundam..

[46] Wang S, Javadpour F, Feng Q (2016). Confinement correction to mercury intrusion capillary pressure of shale nanopores. Sci. Rep-UK.

[47] Espinoza DN, Santamarina JC (2010). Water-CO_2_-mineral systems: Interfacial tension, contact angle, and diffusion—implications to CO_2_ geological storage. Water Resour. Res..

[48] Wang L, Chen Z, Wang C, Elsworth D, Liu W (2019). Reassessment of coal permeability evolution using steady-state flow methods: The role of flow regime transition. Int. J. Coal Geol..

[49] Sander R, Pan Z, Connell LD (2017). Laboratory measurement of low permeability unconventional gas reservoir rocks: A review of experimental methods. J. Nat. Gas Sci. Eng..

[50] Tsang YW (1984). The effect of tortuosity on fluid flow through a single fracture. Water Resour. Res..

[51] Witherspoon PA, Wang JS, Iwai YK, Gale JE (1980). Validity of cubic law for fluid flow in a deformable rock fracture. Water Resour. Res..

